# Characteristics of Hospitalized COVID-19 Patients Discharged and Experiencing Same-Hospital Readmission — United States, March–August 2020

**DOI:** 10.15585/mmwr.mm6945e2

**Published:** 2020-11-13

**Authors:** Amy M. Lavery, Leigh Ellyn Preston, Jean Y. Ko, Jennifer R. Chevinsky, Carla L. DeSisto, Audrey F. Pennington, Lyudmyla Kompaniyets, S. Deblina Datta, Eleanor S. Click, Thomas Golden, Alyson B. Goodman, William R. Mac Kenzie, Tegan K. Boehmer, Adi V. Gundlapalli

**Affiliations:** 1 CDC COVID-19 Response Team.

Coronavirus disease 2019 (COVID-19) is a complex clinical illness with potential complications that might require ongoing clinical care (*1*–*3*). Few studies have investigated discharge patterns and hospital readmissions among large groups of patients after an initial COVID-19 hospitalization (*4*–*7*). Using electronic health record and administrative data from the Premier Healthcare Database,* CDC assessed patterns of hospital discharge, readmission, and demographic and clinical characteristics associated with hospital readmission after a patient’s initial COVID-19 hospitalization (index hospitalization). Among 126,137 unique patients with an index COVID-19 admission during March–July 2020, 15% died during the index hospitalization. Among the 106,543 (85%) surviving patients, 9% (9,504) were readmitted to the same hospital within 2 months of discharge through August 2020. More than a single readmission occurred among 1.6% of patients discharged after the index hospitalization. Readmissions occurred more often among patients discharged to a skilled nursing facility (SNF) (15%) or those needing home health care (12%) than among patients discharged to home or self-care (7%). The odds of hospital readmission increased with age among persons aged ≥65 years, presence of certain chronic conditions, hospitalization within the 3 months preceding the index hospitalization, and if discharge from the index hospitalization was to a SNF or to home with health care assistance. These results support recent analyses that found chronic conditions to be significantly associated with hospital readmission (*6*,*7*) and could be explained by the complications of underlying conditions in the presence of COVID-19 (*8*), COVID-19 sequelae (*3*), or indirect effects of the COVID-19 pandemic (*9*). Understanding the frequency of, and risk factors for, readmission can inform clinical practice, discharge disposition decisions, and public health priorities such as health care planning to ensure availability of resources needed for acute and follow-up care of COVID-19 patients. With the recent increases in cases nationwide, hospital planning can account for these increasing numbers along with the potential for at least 9% of patients to be readmitted, requiring additional beds and resources.

Data for this study were obtained from the Premier Healthcare Database, which includes discharge records from 865 nongovernmental, community, and teaching hospitals that contributed inpatient data during the study period. COVID-19 patients were identified through *International Classification of Diseases, Tenth Revision, Clinical Modification* (ICD-10-CM) discharge diagnosis code of U07.1 (COVID-19, virus identified) during April–July 2020 or B97.29 (Other coronavirus as the cause of disease classified elsewhere [recommended before the April 2020 release of U07.1]^†^) during March–April 2020. Both codes were used for discharges during April. The patient’s first hospitalization with a COVID-19 discharge diagnosis was defined as the index hospitalization. Any subsequent hospitalization occurring within 2 months of the index hospitalization discharge date through August 2020, whether for COVID-19 or other health complications, was considered a hospital readmission.^§^ Hospital readmissions that occurred >2 months after the index hospitalization were excluded. In the Premier Healthcare Database, readmissions were only recorded if a patient returned to the same hospital where the index hospitalization occurred.

Demographic and clinical characteristics of patients at their index hospitalization were compared regarding discharge disposition and readmission status (none versus one or more). Presence of selected chronic conditions associated with a more severe COVID-19 clinical course were identified through ICD-10-CM diagnosis codes during or before the index COVID-19 hospitalization. Visits before the index hospitalization included all inpatient encounters for the cohort during calendar year 2020 only. Five chronic conditions that have been identified by CDC to increase or possibly increase the risk for severe COVID-19–associated illness (chronic obstructive pulmonary disease, heart failure, diabetes [type 1 or type 2, with chronic complications], chronic kidney disease, and obesity [body mass index ≥30 kg/m^2^], including severe obesity, [body mass index ≥40 kg/m^2^]) were mapped to ICD-10-CM codes using the Elixhauser Comorbidity Index (a method for classifying comorbidities based on ICD diagnosis codes found in administrative data; each comorbidity category is dichotomous [present or absent]) and implemented with the Elixhauser Comorbidity Software for ICD-10-CM (beta version; Agency for Healthcare Research and Quality) and R software (version 4.0.92020; The R Foundation)^¶^ (*10*). The following three clinical severity indicators were defined using hospital chargemaster records (i.e., the comprehensive list of all items billable to a hospital patient or to a patient’s insurance provider): intensive care unit (ICU) admission, invasive mechanical ventilation, and noninvasive ventilation. Time to readmission after the index hospitalization was calculated as the difference in days between date of readmission and date of discharge from the previous hospitalization. The primary discharge diagnosis for each hospitalization was categorized into Clinical Classification Software Refined Categories to approximate the primary reason for the hospital stay. A multivariable generalized estimating equation model assessed the odds of readmission, accounting for within-facility correlation. Covariates included in the model were age, sex, race/ethnicity, presence of selected chronic conditions, discharge disposition category, and clinical severity indicators. This activity was reviewed by CDC and was conducted consistent with applicable federal law and CDC policy.**

During March–July 2020, a total of 126,137 patients within the Premier Healthcare Database were hospitalized for COVID-19. The majority of patients were admitted from a non–health care setting (81%), followed by transfer from another hospital, clinic, or SNF (18%) (Table 1). During the index hospitalization, 15% of patients were admitted to an ICU, 13% required invasive mechanical ventilation, and 4% required noninvasive ventilation. At the time of the index hospitalization or at any time during 2020 before the hospitalization, 62% of patients had an ICD-10-CM diagnosis code for one or more of the following five chronic conditions: chronic obstructive pulmonary disease (21%), heart failure (16%), diabetes mellitus type 1 or type 2 (27%), chronic kidney disease (21%), or obesity (27%). Overall, 10,008 (8%) patients had been hospitalized at the same hospital in the 3 months preceding their index COVID-19 hospitalization. Approximately 15% of patients (19,594) died during the index hospitalization.

**TABLE 1:** Demographic characteristics of hospitalized COVID-19 patients at index hospitalization, by readmission status — Premier Healthcare Database, United States, March–August 2020

Characteristic	No. (%)		
	Total	Not readmitted	Readmitted at least once
	N = 126,137	N = 116,633	N = 9,504
**Age group (yrs)**			
<18	1,170 (0.9)	1,095 (0.9)	75 (0.8)
18–39	16,699 (13.2)	15,741 (13.5)	958 (10.1)
40–49	14,490 (11.5)	13,674 (11.7)	816 (8.6)
50–64	35,451 (28.1)	32,923 (28.2)	2,528 (26.6)
65–74	25,419 (20.2)	23,250 (19.9)	2,169 (22.8)
75–84	19,864 (15.7)	18,061 (15.5)	1,803 (19.0)
≥85	13,044 (10.3)	11,889 (10.2)	1,155 (12.2)
**Race/Ethnicity**			
Asian, non-Hispanic	3,652 (2.9)	3,429 (2.9)	223 (2.4)
Black, non-Hispanic	29,226 (23.2)	26,819 (23.0)	2,407 (25.3)
Hispanic	26,921 (21.3)	25,412 (21.8)	1,509 (15.9)
White, non-Hispanic	49,133 (39.0)	44,807 (38.4)	4,326 (45.5)
Other	13,048 (10.3)	12,194 (10.5)	854 (9.0)
**Sex**			
Female	60,426 (47.9)	55,827 (47.9)	4,599 (48.4)
Male	65,597 (52.0)	60,695 (52.0)	4,902 (51.6)
Unknown	114 (0.1)	111 (0.1)	—*
**Point of origin**			
Non–health care	102,482 (81.2)	94,796 (81.3)	7,686 (74.5)
Clinic	6,787 (5.4)	6,217 (5.3)	570 (5.5)
Transfer from a different hospital	8,425 (6.7)	7,968 (6.8)	457 (4.4)
Transfer from SNF or ICF	5,940 (4.7)	5,324 (4.6)	616 (6.0)
Transfer from health facility	1,437 (1.1)	1,339 (1.1)	98 (1.0)
Court/Law enforcement	252 (0.2)	241 (0.2)	11 (0.1)
Born inside the hospital	45 (0.0)	44 (0.0)	—*
Not available	441 (0.3)	410 (0.4)	31 (0.3)
**U.S. Census division**			
East North Central	16,009 (12.7)	14,547 (12.5)	1,462 (15.4)
East South Central	5,986 (4.7)	5,544 (4.8)	442 (4.7)
Middle Atlantic	39,673 (31.5)	36,456 (31.3)	3,217 (33.9)
Mountain	8,852 (7.0)	8,355 (7.2)	497 (5.2)
New England	3,768 (3.0)	3,346 (2.9)	422 (4.4)
Pacific	6,511 (5.2)	6,138 (5.3)	373 (3.9)
South Atlantic	27,407 (21.7)	25,683 (22.0)	1,724 (18.1)
West North Central	4,364 (3.5)	3,998 (3.4)	366 (3.9)
West South Central	13,567 (10.8)	12,566 (10.8)	1,001 (10.5)

**Abbreviations:** COVID-19 = coronavirus disease 2019; ICF = intermediate care facility; SNF = skilled nursing facility.

* Cell sizes <10 were suppressed.

Among the 106,543 patients discharged from the index admission, 9,504 (9%) were readmitted, including 1,667 (1.6%) who were readmitted more than once. The median interval from discharge to first readmission was 8 days (interquartile range = 3–20 days). Less than 0.1% of patients died during readmission (data suppressed for privacy).

Among all patients who were discharged after the index hospitalization, 60% were discharged to home or self-care (to home without any additional professional services provided such as home nursing health care), 15% to a SNF, 10% to home with assistance from a home health organization, 4% to hospice, 4% to ongoing care, and 5% to other locations (Table 2). Readmission was more common among patients discharged to a SNF (15%) or with home health organization support (12%), compared with patients discharged to home or self-care (7%). Median age, severity markers, time to readmission and length of stay differed by index hospitalization discharge disposition category.

**TABLE 2:** Discharge status and subsequent readmissions among 126,137 COVID-19 patients* with an index hospitalization — United States, March–August 2020

Characteristic	Location to which patient was discharged from index hospitalization					
	Home or self-care	SNF	Home health organization	Hospice	Ongoing care^†^	Other^§^
**Discharged (N = 106,543 [85%])**						
No. of patients discharged, (%)	64,475 (60)	16,339 (15)	12,223 (10)	3,807 (4)	4,404 (4)	5,295 (5)
Length of index hospitalization, days, median (IQR)	4 (2–7)	8 (5–15)	8 (4–14)	7 (4–12)	16 (7–29)	3 (1–7)
Male, %	51	47	49	47	57	61
Median age, yrs	53	76	68	83	66	61
≥1 chronic condition, %	53	72	70	67	70	57
ICU admission, %	35	42	45	53	63	42
**Readmitted (N = 9,504, 9%)** ^¶^						
No. (%) of patients readmitted	4,406 (7)	2,517 (15)	1,469 (12)	136 (4)	494 (11)	482 (9)
No. days to readmission, median (IQR)	7 (3–17)	11 (5–25)	8 (3–19)	0 (0–3)	10 (3–25)	6 (1–21)
Length of hospitalization, days, median (IQR)	4 (2–7)	6 (3–9)	5 (3–8)	3 (1–6)	6 (3–10)	4 (2–8)
Male, %	51	50	49	51	62	64
Median age, yrs	58	75	72	80	67	59
≥1 chronic condition, %	67	80	80	75	77	67

**Abbreviations:** COVID-19 = coronavirus disease 2019; ICU = intensive care unit; IQR = interquartile range; SNF = skilled nursing facility.

* A total of 19,594 (15%) patients died during the index hospitalization; 59% of decedents were male, median age was 74 years, 75% had one or more chronic conditions, the median hospitalization duration was 8 days (IQR = 4–15 days), and 68% of patients were admitted to an ICU.

^†^ Ongoing care categories include discharged/transferred to cancer center, admitted as an inpatient to this hospital, still a patient, discharged/transferred to federal hospital, discharged/transferred to swing bed unit (a unit within an acute care hospital where patients receive the same skilled level of care that is available at skilled nursing facilities), discharged/transferred to another rehabilitation facility, discharged/transferred to long-term care hospitals that provide acute inpatient care with an average length of stay of ≥25 days, discharged to a psychiatric hospital, discharged/transferred to a critical access hospital.

^§^ Other category includes patients who were discharged to other facilities and those who left against medical advice.

^¶^ Readmitted from discharged location noted in column (after index hospitalization).

When controlling for covariates, the odds of readmission increased with the presence of chronic obstructive pulmonary disease (OR = 1.4), heart failure (OR = 1.6), diabetes (OR = 1.2), and chronic kidney disease (OR = 1.6). Patients were more likely to be readmitted if they had been discharged from the index hospitalization to a SNF (OR = 1.4) or with home health organization support (OR = 1.3) than if they had been discharged to home or self-care. Compared with persons aged 18–39 years, the odds of readmission increased with age among persons aged ≥65 years (Table 3). Adjusted odds of readmission of patients with a hospitalization in the 3 months preceding their index hospitalization were 2.6 times the odds of those who were not hospitalized in the preceding 3 months. Non-Hispanic White persons were more likely to be readmitted than were those of other racial/ethnic groups. Common primary discharge diagnoses after readmission were infectious and parasitic diseases (primarily COVID-19; 45%) and diseases of the circulatory (11%) and digestive (7%) systems (Supplementary Table, https://stacks.cdc.gov/view/cdc/96391).

**TABLE 3:** Generalized estimating equation model showing the adjusted odds of readmission among persons hospitalized with COVID-19 — United States, March–August 2020

Characteristic	Odds ratio (95%CI)	Standard error*	P-value
**Age group (yrs), (referent = 18–39 yrs)**			
<18	1.03 (0.80–1.33)	0.13	0.806
40**–**49	0.94 (0.84–1.04)	0.05	0.204
50**–**64	1.08 (0.99–1.17)	0.04	0.078
65**–**74	1.22 (1.12–1.34)	0.05	<0.001
75**–**84	1.32 (1.20–1.46)	0.05	<0.001
≥85	1.37 (1.23–1.53)	0.06	<0.001
**Race/Ethnicity (referent = White, non-Hispanic)**			
Asian, non-Hispanic	0.82 (0.71–0.95)	0.07	0.007
Black, non-Hispanic	0.90 (0.85–0.95)	0.03	<0.001
Hispanic	0.75 (0.71–0.81)	0.03	<0.001
Other	0.80 (0.74–0.87)	0.04	<0.001
**Sex (referent = male)**			
Female	0.94 (0.90–0.99)	0.02	0.015
**Chronic conditions**			
COPD	1.35 (1.28–1.42)	0.03	<0.001
Heart failure	1.58 (1.48–1.67)	0.03	<0.001
Diabetes	1.21 (1.14–1.28)	0.03	<0.001
Chronic kidney disease	1.64 (1.55–1.74)	0.03	<0.001
Obesity	0.95 (0.90–1.00)	0.03	0.049
**Previous hospitalization^†^ (yes versus no)**	2.61 (2.45–2.78)	0.03	<0.001
**Severity measures at index hospitalization**			
Length of stay, days	0.99 (0.99–1.00)	0.00	0.001
ICU admission	0.94 (0.89–0.99)	0.03	0.014
Mechanical ventilation	1.15 (1.04–1.27)	0.05	0.006
Noninvasive ventilation	0.86 (0.81–0.90)	0.03	<0.001
**Discharge category from index hospitalization (referent = home/self-care)**			
SNF	1.37 (1.29–1.47)	0.03	<0.001
Home health organization	1.30 (1.21–1.39)	0.04	<0.001
Hospice	0.24 (0.20–0.29)	0.09	<0.001
Ongoing care	1.22 (1.09–1.36)	0.06	0.001

**Abbreviations:** CI = confidence interval; COPD = chronic obstructive pulmonary disease; COVID-19 = coronavirus disease 2019; ICU = intensive care unit; SNF = skilled nursing facility.

* Standard error of coefficient.

^†^ Patients who had a hospitalization within 3 months before their COVID-19 index hospitalization.

## Discussion

In a cohort of 106,543 patients discharged after an index COVID-19 hospitalization, 9% experienced at least one readmission to the same hospital within 2 months of discharge. More than one readmission occurred in 1.6% of cases. In this analysis, the odds of hospital readmission increased with age among persons aged ≥65 years, presence of one of five selected chronic conditions, hospitalization within the 3 months preceding the index hospitalization, and if discharge from the index hospitalization was to a SNF or to home with health care assistance. Although the proportions of patients in the Premier Healthcare Database cohort who were non-Hispanic Black (23%) or Hispanic (21%) were higher than those proportions in the U.S. Census (13% and 18%, respectively), their odds of readmission were lower than those of non-Hispanic White patients. The slight association of readmission with lengths of stay for hospitalized COVID-19 patients merits further study.

These results are comparable to those of recently published analyses, which found a similar group of chronic conditions to be significantly associated with hospital readmission (*6*,*7*) and could be explained by the complications of underlying conditions in the presence of COVID-19 (*8*), COVID-19 sequelae (*3*), or indirect effects of the COVID-19 pandemic (*9*). Although only a small proportion of patients discharged to home or self-care were readmitted, 7% returned to the hospital within a median of 7 days. One explanation for their readmission is that approximately two thirds of these 4,406 patients had one or more of the selected chronic conditions.

After hospitalization for COVID-19, the most common primary discharge diagnoses from hospital readmission were diseases of the circulatory, digestive, or respiratory systems. Future work will examine the detailed diagnoses recorded during readmissions to better understand COVID-19 sequelae or health conditions that require extended or ongoing care.

The findings in this report are subject to at least five limitations. First, COVID-19 diagnoses were determined by ICD-10-CM, not through laboratory confirmation, potentially leading to misclassification of cases. Second, chronic conditions were identified using ICD-10-CM diagnostic codes used at the index hospitalization or a previous encounter. If a patient had a chronic condition but the condition was not assigned a diagnostic code, that condition would not be recorded in this analysis. Third, primary discharge diagnosis was used to infer the primary reason for hospital admission; other diagnoses might have contributed to the reason for index admission and readmissions. Fourth, patients who received care at different hospitals would not be assessed longitudinally. Finally, the sequelae of COVID-19 could not be completely described among hospitalized patients or among those readmitted. Sequelae might be experienced by patients who are never readmitted to a hospital.

Information on the frequency of, and risk factors for, readmission can inform clinical practice and discharge disposition decisions especially with regard to the acuity and location of ongoing care needed for persons who might appear stable at discharge. Further, addressing priorities such as health care planning to ensure adequate health care resources for acute and post-acute follow-up care of COVID-19 patients is critical at a local, regional, and national level. With the recent increase in cases nationwide, hospital planning can account for these increasing numbers along with the potential for at least 9% of patients to be readmitted, requiring additional beds and resources. Continued public health messaging and interventions to prevent COVID-19 among older persons and those with underlying medical conditions is essential.

**Summary**

**What is already known about this topic?**

Evidence suggests that potential health complications after COVID-19 illness might require ongoing clinical care.

**What is added by this report?**

After discharge from an initial COVID-19 hospitalization, 9% of patients were readmitted to the same hospital within 2 months of discharge. Multiple readmissions occurred in 1.6% of patients. Risk factors for readmission included age ≥65 years, presence of certain chronic conditions, hospitalization within the 3 months preceding the first COVID-19 hospitalization, and discharge to a skilled nursing facility or with home health care.

**What are the implications for public health practice?**

Understanding frequency of, and potential reasons for, readmission after a COVID-19 hospitalization can inform clinical practice, discharge disposition decisions, and public health priorities, such as health care resource planning.

## Footnotes

* The Premier Healthcare Database includes discharge records for adult and pediatric patients from >1,000 nongovernmental, teaching and community hospitals representing approximately 25% of U.S. hospital admissions. Data for this study represented a subset of 865 medical facilities that contributed inpatient encounters to the Premier Healthcare Database during March–August 2020. https://www.premierinc.com/newsroom/category/education/page/2.

† https://www.cdc.gov/nchs/data/icd/Announcement-New-ICD-code-for-coronavirus-3-18-2020.pdf.

§ Two months was twice the period used by the Centers for Medicare & Medicaid Services for unplanned readmission measures as knowledge of COVID-19 has been evolving regarding acute and chronic sequelae.

¶ https://www.cdc.gov/coronavirus/2019-ncov/need-extra-precautions/people-with-medical-conditions.html.

** 45 C.F.R. part 46, 21 C.F.R. part 56; 42 U.S.C. Sect. 241(d); 5 U.S.C. Sect. 552a; 44 U.S.C. Sect. 3501 et seq.
